# Automated EEG Background Analysis and 2-Year Outcomes in Neonatal Hypoxic-Ischemic Encephalopathy

**DOI:** 10.1001/jamanetworkopen.2025.48321

**Published:** 2025-12-16

**Authors:** Marie-Coralie Cornet, Adam L. Numis, Courtney J. Wusthoff, Danilo Bernardo, Ulrike Mietzsch, Cameron Thomas, Niranjana Natarajan, Kaashif A. Ahmad, Aaron Scheffler, Sandra E. Juul, Saeed Montazeri Moghadam, Yvonne W. Wu, Hannah C. Glass

**Affiliations:** 1 Division of Neonatology, Department of Pediatrics, University of California, San Francisco; 2 Department of Neurology and the Weill Institute for Neurosciences, University of California, San Francisco; 3 Department of Neurology, University of California, Davis, Sacramento, California; 4 Division of Neonatology, Department of Pediatrics, Seattle Children’s Hospital, University of Washington School of Medicine, Seattle; 5 Department of Pediatrics, University of Cincinnati College of Medicine, Cincinnati, Ohio; 6 Division of Neurology, Cincinnati Children’s Hospital Medical Center, Cincinnati, Ohio; 7 Division of Pediatric Neurology, Department of Neurology, University of Washington, Seattle; 8 Pediatrix Neonatology of San Antonio, San Antonio, Texas; 9 Department of Epidemiology and Biostatistics, University of California, San Francisco; 10 Baby Brain Activity Center, Pediatric Research Center, New Children’s Hospital, Helsinki University Hospital, Helsinki, Finland; 11 Departments of Physiology and Clinical Neurophysiology, University of Helsinki, Helsinki, Finland

## Abstract

**Importance:**

Hypoxic-ischemic encephalopathy (HIE) remains an important contributor to neonatal mortality and morbidity despite therapeutic hypothermia. Accurate early prognostication of outcomes is essential for clinical management and risk stratification in future trials.

**Objective:**

To evaluate the feasibility and estimative ability of automated electroencephalographic (EEG) background analysis in projecting neurodevelopmental outcomes in neonates with HIE using the Brain State of the Newborn (BSN) score.

**Design, Setting, and Participants:**

This cohort study was a secondary analysis of the High-Dose Erythropoietin for Asphyxia and Encephalopathy (HEAL) trial, which enrolled infants born between January 25, 2017, and October 9, 2019, and were followed up at age 2 years (between age 22 and 36 months). Nine academic centers across the US provided raw EEG data starting within the first 24 hours of life. The data were analyzed between August 15, 2024, and August 25, 2025.

**Main Outcomes and Measures:**

The primary outcome was death or severe neurodevelopmental impairment (NDI) at age 2 years. The BSN scores were computed using a cloud service and correlated with expert reader interpretations. Generalized linear mixed models were used to assess the estimative capabilities (quantified via cross-validated area under the receiver operating characteristic curve [AUROC]) of clinical variables and BSN score for severe NDI or death.

**Results:**

Among 500 infants enrolled in the HEAL trial, 203 were included in the current analysis (median [IQR] gestational age, 39.3 [38.0-40.3] weeks; 121 male [59.6%]). Of these infants, 21 (10.3%) experienced severe NDI, and 28 (13.8%) died. The BSN scores correlated with expert reader EEG background classifications (Pearson correlation coefficient for median BSN, 0.69; 95% CI, 0.64-0.73). Adding median overall BSN score (AUROC, 0.90; 95% CI, 0.84-0.97) or median BSN score at all time points (AUROC, 0.93; 95% CI, 0.88-0.98) significantly improved the prognosis of severe NDI or death compared with clinical variables alone (AUROC, 0.79; 95% CI, 0.70-0.87). Prognostic accuracy of median BSN score overall was similar to expert assessments of EEG background (AUROC, 0.90; 95% CI, 0.81-0.98).

**Conclusions and Relevance:**

These findings suggest that automated EEG background analysis may provide an objective method for early prognostication in neonates with HIE. The BSN scores correlated with expert classifications and may aid in risk stratification. The BSN’s role in the treatment of neonates with HIE in environments lacking expert EEG interpretation requires further study.

## Key Points

**Question:**

Can automated electroencephalographic (EEG) background analysis estimate 2-year neurodevelopmental outcomes in neonates with hypoxic-ischemic encephalopathy?

**Findings:**

In this cohort study of 203 neonates from the High-Dose Erythropoietin for Asphyxia and Encephalopathy trial, the Brain State of the Newborn score estimated neurodevelopmental outcomes, even after only 2 hours of recording. The Brain State of the Newborn score added information to clinical variables and was associated with similar prognostic accuracy as EEG background assessment by neurophysiologists.

**Meaning:**

The findings suggest that early automated EEG background quantification may be a valuable tool for assessing early hypoxic-ischemic encephalopathy severity, identifying neonates for inclusion in studies of adjuvant neuroprotective strategies, and providing family counseling.

## Teaser

This cohort study evaluates the feasibility and estimative ability of automated electroencephalographic (EEG) background analysis in the prognosis of neurodevelopmental outcomes in infants with hypoxic-ischemic encephalopathy.

## Introduction

Perinatal hypoxic-ischemic encephalopathy (HIE) affects 1 to 3 per 1000 infants in high-income countries and substantially contributes to neonatal mortality and morbidity worldwide.^1^ Despite therapeutic hypothermia, 30% to 50% of neonates with HIE either die or develop neurodevelopmental impairment (NDI).^2^ Accurate prognostication of death or severe NDI is crucial for guiding clinical management, research into future neuroprotective strategies, and family communication.^3,4^ Various biological, clinical, neurophysiologic, and imaging risk factors have been associated with outcomes in neonates with HIE. Electroencephalographic (EEG) background assessment in the days following birth provides a strong estimation of neurodevelopmental outcomes.^5,6,7^ The failure of EEG background to normalize within a specific time frame was first identified as a risk factor for adverse outcomes by Sarnat and Sarnat in 1976.^8^ However, EEG background evaluation is time consuming; depends on expertise; and, except for EEG background amplitude and continuity, is subject to high interrater variability.^6,7,9^

Machine learning advances have enabled the development of automated EEG background grading systems,^10,11,12^ offering objective, scalable, and reproducible interpretations of EEG data.^12,13,14^ However, most tools developed for neonatal EEG analysis were validated only on single-center data, often with collaboration and expertise from the research teams who created them, or used only selected artifact-free EEG signals.^14,15,16,17^ Recently, Montazeri et al^18^ introduced the Brain State of the Newborn (BSN) score, which is derived from deep learning–based EEG classifiers, to quantify EEG background in infants with HIE. The BSN score, ranging from 0 to 100, correlates well with human expert classification.^18^ Despite its promise, few centers have used this algorithm for outcome prognostication,^3,14,16,19^ and its practicality and performance in a multicenter cohort of neonates with noncurated EEG remains untested.

The High-Dose Erythropoietin for Asphyxia and Encephalopathy (HEAL) trial was a multicenter, randomized clinical trial that investigated the neuroprotective effects of erythropoietin in infants with moderate and severe HIE undergoing therapeutic hypothermia.^2^ Nine of 17 sites in the HEAL trial submitted raw EEG tracings as part of the HEAL-EEG substudy. Here, we aimed to evaluate the feasibility of using automated EEG analysis across the HEAL-EEG centers. We assessed the correlation between expert human EEG background evaluation and BSN scores and the estimative ability of BSN for 2-year outcomes using data available 2 hours after EEG initiation and 24, 48, and 72 hours after birth.

## Methods

This cohort study is a secondary analysis of the HEAL trial.^2,20^ This study was approved by all study site institutional review boards (including 2 sites in California, 1 in the District of Columbia, 1 in Indiana, 2 in Ohio, 1 in Pennsylvania, 1 in Texas, and 1 in Washington). Parents provided written informed consent. The study followed the Strengthening the Reporting of Observational Studies in Epidemiology (STROBE) reporting guideline.

Infants born between January 25, 2017, and October 9, 2019, were enrolled in the HEAL trial if they met all inclusion criteria for presumed HIE: (1) gestational age 36 weeks or older; (2) perinatal depression evidenced by Apgar score less than 5 at 10 minutes, cardiorespiratory depression beyond age 10 minutes, pH less than 7.0, or base deficit of at least 15 mmol/L within 60 minutes of birth; (3) moderate to severe neonatal encephalopathy based on a modified Sarnat examination between age 1 and 6 hours; and (4) treatment with therapeutic hypothermia. Demographic characteristics and clinical variables were collected in the parent trial as described previously.^2^ Maternal race (Asian, Black, White, multiracial, or other [including American Indian or Alaska Native, Native Hawaiian or Other Pacific Islander, unknown, and not reported]) and ethnicity (Hispanic or non-Hispanic) were extracted from the HEAL database. Nine of the 17 HEAL sites completed continuous EEG during cooling and provided raw EEG tracings for centralized analysis.^21^ We included neonates with more than 24 hours of raw EEG data, starting before age 24 hours. Continuous EEG was acquired with at least 8 cerebral electrodes using 10- to 20-electrode placement modified for the neonate. Two board-certified pediatric clinical neurophysiologists (A.L.N. and C.J.W.), experts in neonatal monitoring, reviewed a subset of 150 EEG recordings from 7 sites, masked to treatment and outcomes as reported in a the previous study.^21^ They independently classified five 60-minute epochs (onset and 24, 36, 48, and 72 hours after birth) of EEG recordings into 3 background categories according to American Clinical Neurophysiology Society recommendations^22^: normal, excessively discontinuous, and severely abnormal. Disagreements were resolved by consensus. The previous study showed substantial interrater agreement (weighted Cohen κ = 0.74).^21^

For automated analysis, anonymized continuous EEG files were converted to an 8-channel bipolar montage and European data format using Persyst, version 14 (Persyst Development LLC) from April 5 to November 31, 2024. The open-access computerized cloud service tool^23^ developed by the Baby Brain Activity (BABA) research center at the University of Helsinki was used to calculate BSN scores, ranging from 0 (inactive EEG) to 100 (fully active) as previously described.^18^ Full EEG data were analyzed without further preprocessing. Artifacts were automatically rejected to maximize clinical utility and validity. In brief, BSN is an interictal measure of EEG background activity. The BSN scores were calculated for each 2-second interval of tracings. The BABA research center then performed a postprocessing step that averages BSN across channels and aggregates them into a median for every 10 minutes of tracing. In this postprocessing step, every 1-minute nonoverlapping segment of the BSN was considered missing if it contained more than 30 seconds of artifact or more than 6 seconds of seizure detection. The analysis algorithm has been previously explained in detail, including its rationale, design, and validations.^14,18^ Minimal, median, and maximal BSN values based on the available 10-minute outputs for each infant were determined for 4 epochs: the first 2 hours of recording and 12 to 24 hours, 36 to 48 hours, and 60 to 72 hours after birth.

The primary outcome was death or severe NDI at age 2 years. The secondary outcome was neurodevelopment on a 5-level ordinal scale of no NDI, mild NDI, moderate NDI, severe NDI, or death. The degree of NDI was defined as the worst result of either the cognitive or motor outcome as prespecified in the parent HEAL trial.^2^ Cognitive outcome was assessed using the Bayley Scales of Infant and Toddler Development, Third Edition cognitive score and categorized as normal (≥90), mild (85-89), moderate (70-84), or severe (<70). For motor outcome, hemiparesis or diparesis with a modified Growth Motor Function Classification Scale (GMFCS) score of less than 1 or no cerebral palsy with a modified GMFCS of 1 was considered mild; quadriparesis with a modified GMFCS score of less than 1, hemiparesis or diparesis with a modified GMFCS score of 2 or less, or no cerebral palsy with a modified GMFCS score of 2 was considered moderate; and quadriparesis with a modified GMFCS score of 1 or greater or hemiparesis, diparesis, or no cerebral palsy with a modified GMFCS score of 3 or greater was considered severe.^2^

### Statistical Analysis

**Figure 1.**
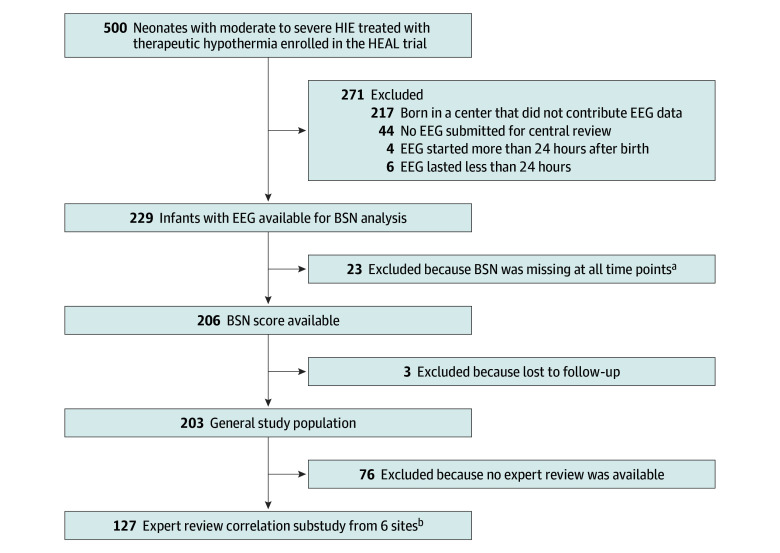
Flow Diagram of Infants Eligible for the Study BSN indicates Brain State of the Newborn; EEG, electroencephalography; HEAL, High-Dose Erythropoietin for Asphyxia and Encephalopathy; HIE, hypoxic-ischemic encephalopathy. ^a^Missing due to a technical issue with the artifact detector in the postprocessing stage. For these infants, the tracing was wrongly considered to have constant artifacts despite a good-quality tracing per expert reviewers. This false artifact detection occurred in 3 different centers, affecting tracings for 11 of 11 infants, 10 of 20 infants, and 2 of 41 infants, respectively. ^b^An expert review was performed for 150 infants as part of the HEAL-EEG study and included 127 infants whose BSN score was available and 23 with missing BSN scores at all time points.

For all statistical analyses, we excluded infants whose BSN score was always missing or who had no follow-up available, unless specified otherwise (Figure 1). We used descriptive statistics to assess the association between clinical and EEG characteristics and severe NDI or death. Pearson correlation coefficients were used to evaluate the correlation between expert reader interpretation of the EEG and BSN scores. We categorized maximal, median, and minimal BSN values during each epoch into 4 categories of increasing BSN values (<25, 25 to <50, 50 to <75, and ≥75) to ease results interpretation and visualization for clinicians. We used the Cochran-Armitage test of trend to assess the increasing frequency of severe NDI or death by BSN category for each epoch. Because maximal, median, and minimal BSN values were correlated and had similar trends, we focused only on median BSN for our projection modeling.

To assess the estimative ability of EEG background assessment for neurodevelopmental outcomes, we built models of increasing complexity, including the following sets of estimators: (1) BSN value at each time point alone, (2) clinical variables (Apgar score at 5 minutes and HIE severity) alone, and (3) combination of clinical variables and BSN values across time points. The primary outcome, binary death or severe NDI at age 2 years, was modeled for each set of estimators via generalized linear mixed models (GLMMs), with a site-specific random intercept to account for correlations within sites. The GLMMs were estimated using the R package lme4,^24^ and receiver operating characteristic (ROC) analyses were conducted using the R package pROC.^25^ Estimated probabilities were obtained via leave-one-site-out cross-validation, in which models were iteratively trained holding 1 site out at a time and then forming estimations for the held-out site. Estimated probabilities for held-out sites were pooled to estimate the area under the ROC curve (AUROC), and 95% CIs were obtained via 2000 bootstrap replicates. The ROC curves were compared using DeLong test, given minimal within-center correlations as assessed via mixed-effects models. While infants with missing BSN scores at all time points were excluded, infants missing BSN scores at 1 to 2 specific time points (eFigure 1 in Supplement 1) were included in all analyses except the analysis using BSN scores at the missing time point (<5% missingness). In a sensitivity analysis, we used multiple imputation using chained equations and 5 imputations to impute missing BSNs in all infants with 2-year follow-up and more than 24 hours of EEG available, starting before 24 hours.

Statistical significance was set at a 2-sided* P* < .05 using χ^2^ test for categorical variables and the Wilcoxon rank sum test for continuous variables. All statistical analyses were performed offline between August 15, 2024, and August 25, 2025, using Python, version 3.11.9 (Python Software Foundation); R, version 4.4 (R Foundation for Statistical Computing); and Stata, version 17 (StataCorp LLC).

## Results

Among 500 infants enrolled in the HEAL trial, 239 were born in a center that submitted EEG tracings and had at least 1 tracing available for centralized review, and 229 had at least 24 hours of EEG recording starting before 24 hours of life available for analysis. Twenty-three infants were excluded due to false detection of continuous artifacts, leading to missing BSN scores in the BABA integrated postprocessing step for the entire duration of the tracing (Figure 1). Three additional infants were excluded due to a lack of follow-up. Hence, 203 infants (median [IQR] gestational age, 39.3 [38.0-40.3] weeks; 82 female [40.4%] and 121 male [59.6%]; maternal race, 20 Asian [9.9%], 26 Black [12.8%], 137 White [67.5%], and 20 multiracial or other [9.9%]; maternal ethnicity, 55 Hispanic [27.1%] and 148 non-Hispanic [72.9%]) from 8 centers, each including between 13 and 40 patients (median [IQR], 25 [18-31] patients), were included in this analysis. Infants included in this study had similar clinical characteristics, HIE severity, and outcomes to infants in the general HEAL cohort (eTable 1 in Supplement 1).

Among these 203 infants, 49 (24.1%) had severe NDI or died. A total of 28 infants (13.8%) died, 21 (10.3%) had severe NDI, 20 (10.3%) had moderate NDI, 25 (12.3%) had mild NDI, and 109 (53.7%) had no NDI. The demographic and EEG characteristics of infants with and without severe NDI or who had died are presented in the Table. As expected, infants with severe NDI or who died (n = 49) compared with those who did not (n = 154) had significantly lower Apgar scores (Apgar at 5 minutes: median [IQR], 3 [1-4] vs 4 [2-5]; Apgar at 10 minutes: median [IQR], 3 [2-5] vs 5 [4-7]) (*P* < .001), worse acidosis (median [IQR] pH, 6.9 [6.7-7.0] vs 7.0 [6.9-7.1]; *P* < .001), and more severe encephalopathy (median Sarnat score [IQR], 15 [12-17] vs 12 [10-13]; *P* < .001). Infants with severe NDI or death also had significantly lower minimal, median, and maximal BSN values at each time point (Table). Individual outcome descriptions of all infants with severe NDI are provided in eTable 2 in Supplement 1.

**Table.** Clinical and EEG Characteristics of 203 Infants With Moderate to Severe HIE, Stratified by 2-Year Outcomes

Characteristic	Total (N = 203)	2-y Outcomes, median (IQR) or No. (%)		*P* value^b^
		Severe or death (n = 49)^a^	No severe NDI or death (n = 154)	
**Demographics**				
Maternal race				
Asian	20 (9.9)	19 (12.3)	1 (2.0)	.09
Black	26 (12.8)	22 (14.3)	4 (8.2)	
White	137 (67.5)	98 (63.6)	39 (79.6)	
Multiracial or other^c^	20 (9.9)	40 (26.0)	15 (30.0)	
Maternal ethnicity				
Hispanic	148 (72.9)	114 (74.9)	34 (69.4)	.52
Non-Hispanic	55 (27.1)	40 (26.0)	15 (30.6)	
**Clinical**				
Gestational age, wk	39.3 (38 to 40.3)	39 (38.1 to 39.9)	39.6 (38 to 40.4)	.28
Sex, No. (%)				
Female	82 (40.4)	21 (42.9)	61 (39.6)	.69
Male	121 (59.6)	28 (57.1)	93 (60.4)	
Apgar at 5 min	3 (2 to 5)	3 (1 to 4)	4 (2 to 5)	<.001
Apgar at 10 min	5 (3 to 6)	3 (2 to 5)	5 (4 to 7)	<.001
Lowest pH	6.9 (6.8 to 7.0)	6.9 (6.7 to 7.0)	7.0 (6.9 to 7.1)	<.001
Worst base excess	−18 (−22 to −14)	−22 (−28 to −18)	−17 (−20 to −13)	<.001
Severe HIE, No. (%)	48 (23.6)	31 (63.2)	17 (11.0)	<.001
Sarnat score	12 (11 to 15)	15 (12 to 17)	12 (10 to 13)	<.001
**EEG**				
Age at EEG initiation, h	8.3 (6.5 to 9.9)	8.4 (7.1 to 9.9)	8.0 (6.5 to 9.9)	.47
Duration of recording, h	83 (74 to 94)	85 (70 to 98)	83 (75 to 92)	.88
EEG background by report, No. (%)^d^				
Normal	87 (42.9)	2 (4.1)	85 (55.2)	<.001
Excessively discontinuous	71 (35.0)	13 (26.5)	58 (37.7)	
Severely abnormal	45 (22.2)	34 (69.4)	11 (7.1)	
**BSN values**				
At onset (n = 203)				
Minimum	48 (31 to 73)	29 (13 to 37)	57 (39 to 79)	<.001
Median	65 (41 to 83)	34 (19 to 51)	74 (57 to 86)	<.001
Maximum	75 (51 to 88)	41 (29 to 57)	81 (65 to 90)	<.001
At 12-24 h of life (n = 196)				
Minimum	47 (28 to 63)	19 (5 to 34)	52 (37 to 65)	<.001
Median	68 (45 to 83)	33 (12 to 48)	75 (61 to 85)	<.001
Maximum	85 (66 to 93)	47 (31 to 70)	89 (79 to 94)	<.001
At 36-48 h of life (n = 199)				
Minimum	51 (31 to 67)	20 (8 to 41)	57 (42 to 72)	<.001
Median	74 (48 to 86)	35 (15 to 55)	78 (66 to 87)	<.001
Maximum	86 (66 to 94)	47 (35 to 68)	90 (83 to 94)	<.001
At 60-72 h of life (n = 183)				
Minimum	50 (32 to 70)	26 (7 to 34)	58 (39 to 72)	<.001
Median	73 (49 to 84)	37 (20 to 56)	79 (66 to 86)	<.001
Maximum	87 (67 to 92)	51 (34 to 70)	88 (81 to 93)	<.001

Abbreviations: BSN, Brain State of the Newborn; EEG, electroencephalography; GMFCS, Gross Motor Function Classification Scale; HIE, hypoxic-ischemic encephalopathy; NDI, neurodevelopmental impairment.

^a^ Severe NDI was defined as Bayley Scales for Infant and Toddler Development, Third Edition cognitive score less than 70, quadriparesis with a modified GMFCS score of 1 or greater, or hemiparesis or diparesis or no cerebral palsy with a modified GMFCS score of 3 or greater per primary High-Dose Erythropoietin for Asphyxia and Encephalopathy trial definitions.

^b^ *P* values were obtained from the χ^2^ test for categorical variables and the Wilcoxon rank sum test for continuous variables.

^c^ Includes American Indian or Alaska Native, Native Hawaiian or Other Pacific Islander, unknown, and not reported.

^d^ The EEG reports from the first day after birth were classified by reviewers masked to clinical history and outcomes as normal continuity, excessively discontinuous, or severely abnormal based on the worst EEG background described in the first 24 hours using previously published pragmatic definitions.^9^

**Figure 2.**
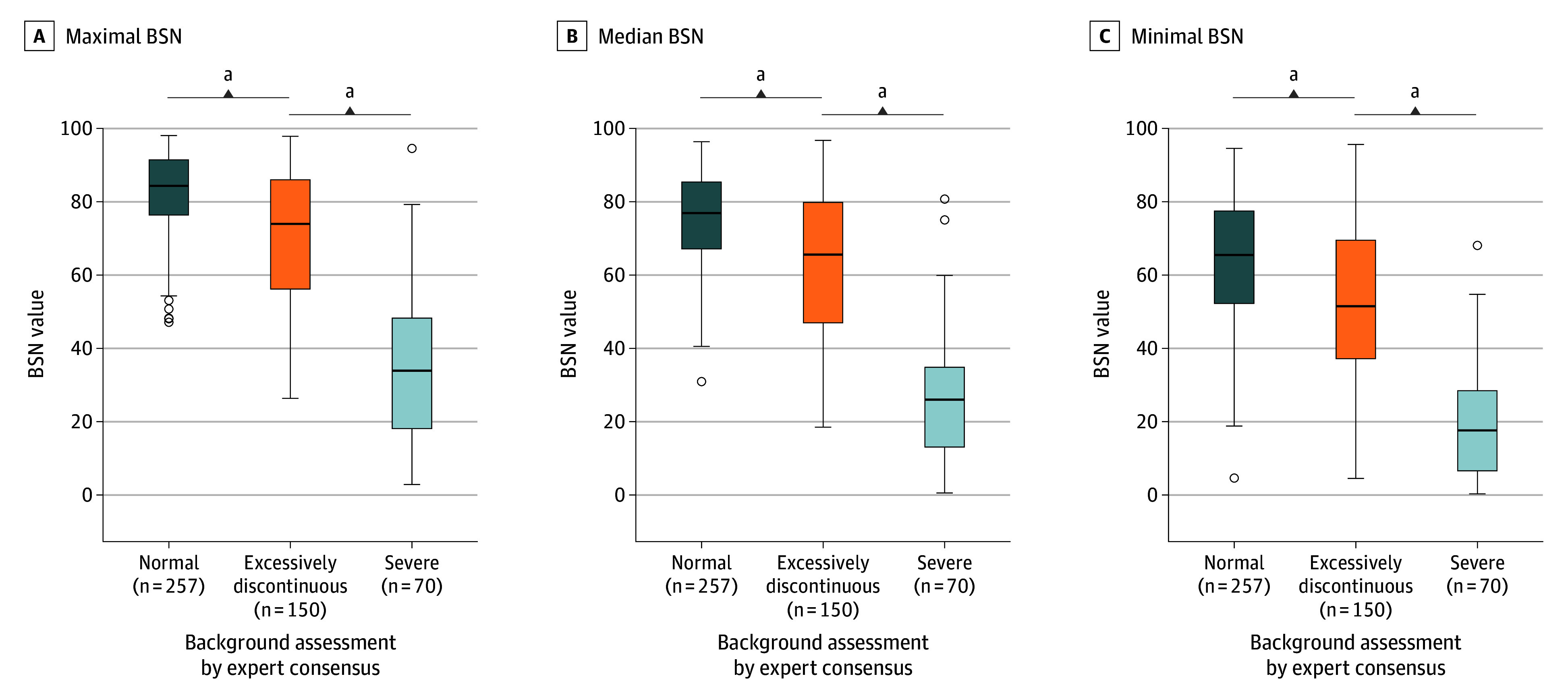
Agreement Between Brain State of the Newborn (BSN) Values and Expert Reader Background Assessments Values included for 123 infants studied at 4 time points (onset and 24, 48, and 72 hours). The numbers for each expert consensus represent the number of EEG background epochs analyzed. For 31 epochs, either the expert consensus (n = 21) or the BSN score (n = 19) was missing. The horizontal bar inside the boxes indicates the median, the lower and upper ends of the boxes are the first and third quartiles, and the whiskers indicate the farthest data point within 1.5 times the IQR of the box edges. Data more extreme than the whiskers are plotted individually as outliers (open circles). ^a^*P* < .001.

Expert human reader interpretation of EEG tracings was available for 127 of the 203 infants (62.6%) as part of the HEAL-EEG study (Figure 1). Pearson correlation coefficients between EEG background by expert consensus and BSN values during the same hour were 0.60 (95% CI, 0.54-0.65) for minimal, 0.69 (95% CI, 0.64-0.73) for median, and 0.70 (95% CI, 0.65-0.75) for maximal BSN scores (Figure 2; eFigure 1 in Supplement 1).

**Figure 3.**
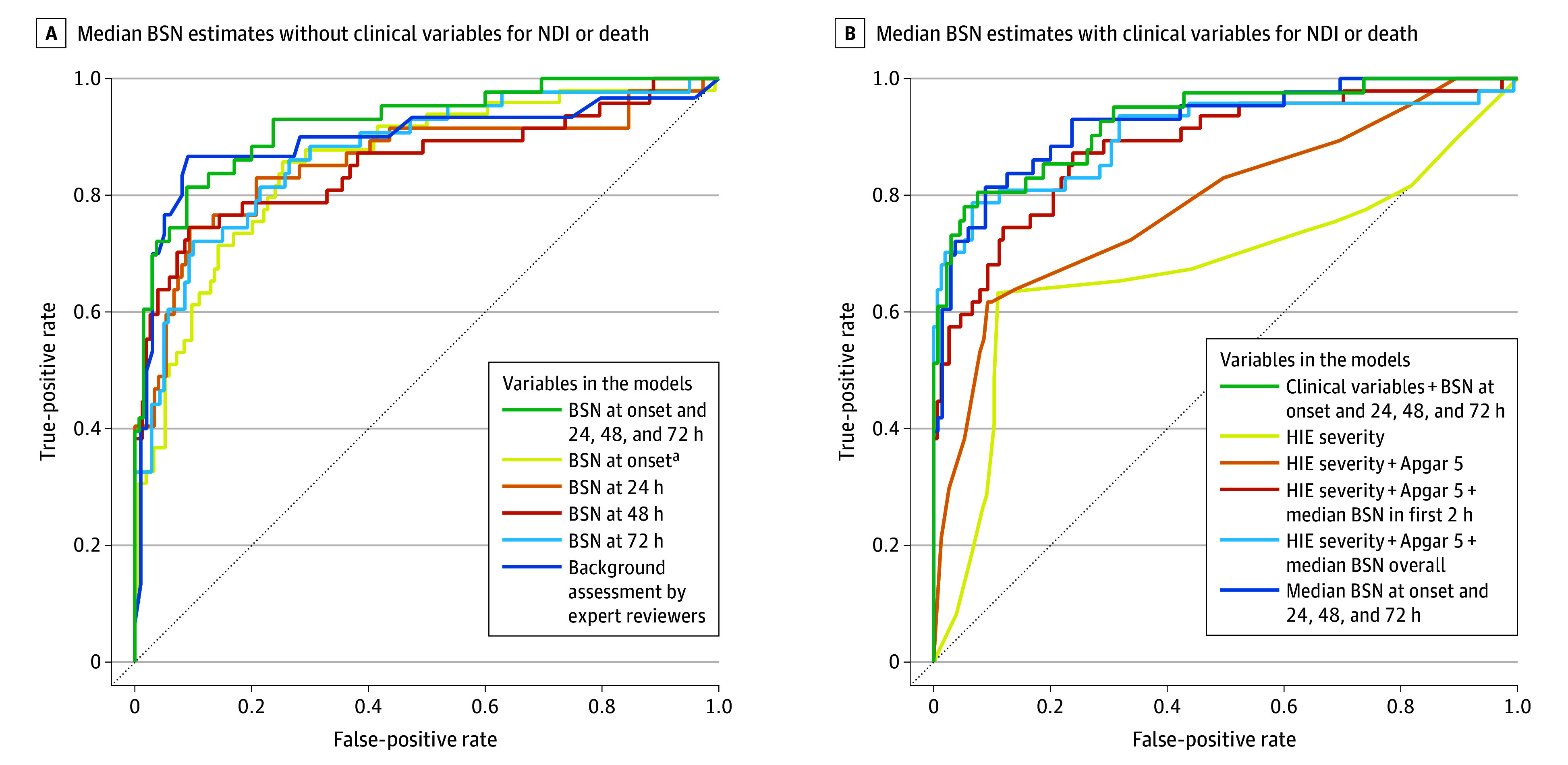
Estimates of Severe Neurodevelopmental Impairment (NDI) or Death by Median Brain State of the Newborn (BSN) Values Alone or Combined With Clinical Variables. All receiver operating characteristic curves were constructed based on the whole cohort (N = 203) except for the EEG background by expert reviewers (n = 127). ^a^Median BSN in the first 2 hours after EEG initiation.

Among the 203 infants included in the cohort, using GLMMS with leave-one-site-out cross-validation, the median BSN at onset had an AUROC of 0.85 (95% CI, 0.79-0.92) for estimating severe NDI or death. The AUROC when combining median BSN at all time points (onset and 24, 48, and 72 hours) was 0.92 (95% CI, 0.87-0.97), which was significantly higher than the median BSN at onset only (0.85; 95% CI, 0.79-0.92) (*P* = .005) as shown in Figure 3A. The AUROC for the overall background assessment by expert readers was 0.90 (95% CI, 0.81-0.98), which was not significantly different from the median BSN at all time points (*P* = .28). The AUROCs for minimal and maximal BSN at all time points are available in eFigure 2 in Supplement 1.

Compared with clinical variables alone (AUROC, 0.79; 95% CI, 0.70-0.87), adding median overall BSN scores (AUROC, 0.90; 95% CI, 0.84-0.97), median BSN scores at onset (AUROC, 0.88; 95% CI, 0.82-0.94), or median BSN scores at all time points (AUROC, 0.92; 95% CI, 0.87-0.97) improved the estimation of severe NDI or death with *P* < .001 by DeLong test comparing clinical variables with BSN plus clinical variables across all 3 comparisons (Figure 3B). However, compared with median BSN at all time points without clinical variables (AUROC, 0.92; 95% CI, 0.87-0.97), adding clinical variables (AUROC, 0.93; 95% CI, 0.88-0.98) did not improve estimation (DeLong test for correlated ROC *P* = .42). The sensitivity analyses with imputed data (for patients missing BSN scores at all time points) found similar values of the AUROC (eTable 3 in Supplement 1).

**Figure 4.**
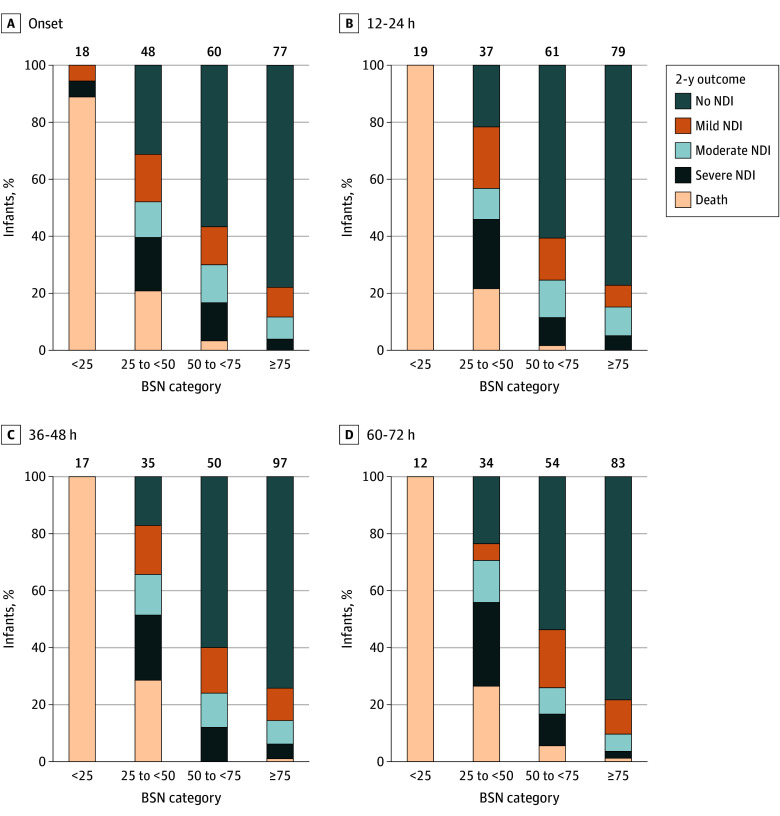
Neurodevelopmental Outcomes Based on Median Brain State of the Newborn (BSN) Score at Each Epoch NDI indicates neurodevelopmental impairment.

Minimal, median, and maximal BSN scores at each time point were associated with the 5-level neurodevelopment or death outcome at age 2 years (Figure 4; eFigure 3 in Supplement 1). For example, among the 18 infants with a median BSN score in the first 2 hours of recording less than 25, 17 (94.4%) either died or had severe NDI compared with only 3 of the 77 infants (3.9%) with a median BSN score of 75 or greater during the same epoch.

## Discussion

This cohort study found that automated EEG background analysis using multicenter data is feasible. The BSN scores correlated with expert EEG background classification at every time point, including in the first 2 hours of recording, and projected 2-year outcomes. The BSN significantly improved prognostication for severe NDI or death compared with clinical variables alone. Hence, information from automated EEG background analysis may potentially improve early prognostication and risk stratification, particularly in centers lacking continuous neonatal EEG expertise.

The EEG background provides a reliable estimation of neurodevelopmental outcomes.^9,21,26,27^ A recent study showed that EEG background continuity described on EEG reports was one of the most predictive features for outcomes in neonates with HIE.^6^ However, EEG background assessment is time consuming, expertise dependent, and often unavailable in real time.^13,28^ Amplitude-integrated EEG (aEEG), displayed in real time at the bedside, is easier to interpret. However, aEEG interpretation also requires training and expertise, and its interpretation is categorical and subjective.^29^ In contrast, BSN provides an objective and continuous measure of EEG background activity. Furthermore, the automated selection of artifact-free epochs suggests that a deep learning model may project outcomes reproducibly, without the need for initial quality assessment of the EEG, with performance similar to that of a human reader. Interestingly, BSN alone without clinical variables showed similar estimates of outcomes as BSN supplemented by clinical variables.

The BSN is provided from the BABA cloud as a granular and fluctuating score for each 2-second interval of tracing, and postprocessing generates an aggregated median for every 10 minutes or 1 hour of tracings, effectively filtering the second-to-second variability of human brain activity. We extracted and used the 10-minute median BSN scores, then aggregated the data over a 2- to 12-hour period. While this technique did not use all the data granularity, it is less influenced by short artifacts and sleep-wake cycles. Furthermore, the variability of the 2-second BSN values may be as challenging to interpret for neonatologists as aEEG. The significant association with outcomes of our aggregated data supports the value of BSN as a reliable estimator of outcomes. Further adjustments and more sophisticated machine learning methods for data postprocessing on the numerical 2-second output may allow for even more precise and accurate outcome projection. For example, a score incorporating time-varying BSN trends information, such as the sleep-wake cycle or seizure activity in addition to BSN, may further enhance the estimative ability of automated EEG background analysis. Ultimately, providing a single numerical EEG score at each time point, incorporating all BSN scores available up to that point, could assist clinicians further in risk stratification and outcome prognostication.

Artifacts are common in neonatal EEG recordings and present substantial challenges for machine learning algorithms. In this study, 23 infants (approximately 10% of those with available EEG data) were excluded, even though their EEGs were rated as high quality by expert reviewers and they had valid 2-second BSN segments. In discussions with the BSN developers, we found that these exclusions were unrelated to the BSN itself. They occurred because erroneous artifact detection during the automated postprocessing phase incorrectly flagged these infants’ BSN scores as missing when generating the 10-minute output using the BABA algorithm. The excluded infants, who were born across 3 different centers, had clinical characteristics comparable to the overall HEAL population, highlighting the need for further refinement of automated EEG processing workflows, including file format and montage harmonization and postprocessing algorithms, to improve clinical applicability.

Maximal, median, and minimal BSN scores for each time point were all highly correlated and associated with neurodevelopmental outcomes. In our estimative models, we chose to focus on the estimative value of the median BSN, as done in other studies using BSN.^15^ Medications given during therapeutic hypothermia, such as morphine and antiseizure medicines, affect EEG background continuity.^30,31^ The maximal BSN score, representing the best average activity in the studied time point, could be less affected by medication and may be valuable in assessing the best activity generated at the time point. However, in this limited sample of infants, we did not see a significant difference in the ability of maximal BSN score to estimate outcome compared with the median BSN score. Future studies could assess the impact of therapeutic hypothermia and sedating medicine on BSN scores and evaluate whether the BSN response to these treatments estimates outcomes.

Most deaths occurred following redirection of care in infants with severe clinical, EEG, and magnetic resonance imaging findings.^32^ Hence, it was essential to confirm that EEG background accuracy in estimating outcome did not reflect a self-fulfilling prophecy. Among infants who survived, those with lower BSN scores had more severe NDI, suggesting that lower BSN values remain a marker of outcomes even when excluding infants in whom the self-fulfilling prophecy may apply. Notably, as in most studies in neonatology, outcome classification was based on neurologic examinations and standardized testing at age 2 years. Severity of NDI is somewhat subjective, and our classification may not represent every child’s or family’s lived experience. Previous studies have shown that clinicians and parents often disagree on what constitutes severe NDI,^33^ which must be acknowledged when using tools such as BSN for parental counseling.

### Limitations

This study had several limitations. First, some BSN values were missing, and it could not be confirmed that the missingness was entirely at random. However, sensitivity analysis using multiple imputations showed similar results when comparing exclusion of the missing data with inclusion using imputation. Most missing data may have stemmed from technical issues during the postprocessing phase of output generation, indicating an opportunity for improvement in this step in future studies. Second, the BSN output is highly complex, allowing for the analysis of the output individually for each channel for every 2 seconds. However, in this proof-of-concept study, we focused on the mean BSN scores across all channels, as suggested by the tool’s authors. Further analysis could evaluate how filtering only some channels or selecting the maximum vs the mean BSN values across channels might improve the BSN’s prognostic ability. Similarly, this analysis focused on the median BSN across all time points rather than using more complex variables to avoid overfitting the data. This process may have led to an underestimation of the potential prognostic ability of BSN. Third, because EEG was started at a median age of 8 hours, when the infant’s body temperature was already low, we could not report on how BSN estimates outcomes in the first 6 hours after birth or in full-term infants with encephalopathy who did not undergo therapeutic hypothermia. Fourth, we did not consider sedating or antiseizure medications in the analysis. These medications may have artificially suppressed the EEG background in some infants. Finally, expert review of EEG tracings was available for only 63% of the included infants, as this study used EEG data previously analyzed in the HEAL-EEG study.^21^ The time-intensive nature of rigorous EEG background scoring by experts limited the number of tracings that could be evaluated. Our convenience sample was selected randomly from patients who had good-quality EEG tracings available for the 72 hours of cooling and throughout rewarming. This sample may be representative of the overall HEAL population, as the clinical characteristics and outcomes were comparable between the reviewed and nonreviewed groups.

## Conclusions

This cohort study found that automated EEG analysis may hold promise for the early and reproducible quantification of EEG background, which might assist clinicians in real-time risk stratification and guide individualized management, especially in centers in which real-time neurophysiology review is not available. Future studies should investigate how BSN projects outcomes before age 6 hours, as well as how therapeutic hypothermia and medications, such as antiseizure medicine and opiates, affect EEG background and BSN prognostic abilities. Additional studies with larger sample sizes are needed to assess whether more complex models could improve outcome projection and how the estimative ability changes if using 2 vs 8 channels of EEG. While there is room for improvement in automating the conversion of EEG files to the correct type and montage before BABA analysis, artifact detection, and postprocessing of the complex BABA cloud output, this study found that the EEG background may be an essential, early, quantifiable, and objective biomarker of neurodevelopmental outcomes. Importantly, because neurodevelopmental outcomes are affected by the home environment,^34,35^ all prognostic tools will remain imperfect at estimating outcomes, leaving room for hope for infants with HIE and their families.

## Notes

Supplement 1. **eTable 1.** Characteristics of All Infants Included in the HEAL Trial Based on Their Inclusion Status in This Study **eTable 2.** Characteristics of Infants Classified as Having Severe NDI **eTable 3.** AUROC for 5 Imputed Datasets, Including all 226 Infants With EEG Processed Through BABA **eFigure 1.** Maximal, Median, and Minimal BSN Values Based on EEG Continuity Scoring by Expert Reviewers at Each Time Point **eFigure 2.** ROC Curves Projecting Severe NDI or Death for Minimal and Maximal BSN Values **eFigure 3.** Neurodevelopmental Outcomes Based on Minimal and Maximal BSN Score at Each Epoch

Supplement 2.
Data Sharing Statement
