# Supplementary material for: Automated EEG Background Analysis and 2-Year Outcomes in Neonatal Hypoxic-Ischemic Encephalopathy
Source: JAMA Netw Open. 2025 Dec 16;8(12):e2548321. doi: 10.1001/jamanetworkopen.2025.48321 (PMC12709380; doi:10.1001/jamanetworkopen.2025.48321)
Supplement: Supplement 1. — eTable 1. Characteristics of All Infants Included in the HEAL Trial Based on Their Inclusion Status in This Study eTable 2. Characteristics of Infants Classified as Having Severe NDI eTable 3. AUROC for 5 Imputed Datasets, Including all 226 Infants With EEG Processed Through BABA eFigure 1. Maximal, Median, and Minimal BSN Values Based on EEG Continuity Scoring by Expert Reviewers at Each Time Point eFigure 2. ROC Curves Projecting Severe NDI or Death for Minimal and Maximal BSN Values eFigure 3. Neurodevelopmental Outcomes Based on Minimal and Maximal BSN Score at Each Epoch [file jamanetwopen-e2548321-s001.pdf]

Supplemental Online Content

Cornet MC, Numis AL, Wusthoff CJ, et al. Automated EEG background analysis and 2-year outcomes in neonatal hypoxic-ischemic encephalopathy. *JAMA Netw Open*. 2025;8(12):e2548321. doi:10.1001/jamanetworkopen.2025.48321

eTable 1. Characteristics of All Infants Included in the HEAL Trial Based on Their Inclusion Status in This Study

eTable 2. Characteristics of Infants Classified as Having Severe NDI

eTable 3. AUROC for 5 Imputed Datasets, Including all 226 Infants With EEG Processed Through BABA

eFigure 1. Maximal, Median, and Minimal BSN Values Based on EEG Continuity Scoring by Expert Reviewers at Each Time Point

eFigure 2. ROC Curves Projecting Severe NDI or Death for Minimal and Maximal BSN Values

eFigure 3. Neurodevelopmental Outcomes Based on Minimal and Maximal BSN Score at Each Epoch

This supplemental material has been provided by the authors to give readers additional information about their work.

© 2025 Cornet MC et al. *JAMA Network Open*.

eTable 1. Characteristics of All Infants Included in the HEAL Trial Based on Their Inclusion Status in This Study

Characteristics	Overall	Excluded Groups			Included Groups (BSN Available)		P value	
Groups	Total N=500	No EEG, started <24hours of age and lasting >24 hours, available N=271	EEG available but no BSN (post-processing artefact) N=23	BSN available but no follow-up N=3	BSN available but no expert review available N=76	BSN available and expert review available N=127	Excluded vs Included	With and without expert review
**Clinical characteristics**								
Gestational Age (weeks)	39.1 (38.0, 40.3)	39.1 (38.1, 40.3)	38.9 (37.3, 40.0)	40.9 (36.9, 41.0)	39.0 (37.9, 40.1)	39.6 (38.3, 40.6)	0.31	0.12
Male sex	275 (55%)	141 (52%)	10 (43%)	3 (100%)	47 (62%)	74 (58%)	0.09	0.62
Apgar 5 minutes	3.0 (2.0, 5.0)	3.0 (2.0, 5.0)	3.0 (2.0, 4.0)	5.0 (4.0, 5.0)	3.0 (2.0, 5.0)	3.0 (2.0, 4.0)	0.98	0.50
Apgar 10 minutes	5.0 (3.0, 7.0)	5.0 (3.0, 7.0)	4.0 (3.0, 5.0)	5.0 (5.0, 6.0)	4.0 (3.0, 6.0)	5.0 (4.0, 6.0)	0.24	0.54
Lowest pH	6.9 (6.8, 7.0)	7.0 (6.8, 7.0)	6.9 (6.8, 7.1)	6.9 (6.6, 7.0)	6.9 (6.8, 7.0)	6.9 (6.8, 7.1)	0.96	0.51
Worst Base excess	-18 (-23, -14)	-18 (-23, -14)	-18 (-22, -14)	-25 (-28, -17)	-19 (-23, -15)	-17 (-22, -13)	0.90	0.2
**Clinical encephalopathy**								
Severe HIE	113 (23%)	56 (21%)	9 (39%)	0 (0%)	24 (32%)	24 (19%)	0.38	0.04
Sarnat Score	12 (11, 14)	12 (10, 14)	13 (11, 16)	12 (11, 13)	13 (11, 15)	12 (11, 14)	0.12	0.10
**Electroencephalography**								
EEG/aEEG background by report[1]							0.24	0.88
Normal	186 (38%)	91 (35%)	7 (30%)	1 (33%)	31 (41%)	56 (44%)		
Excessively discontinuous	176 (35%)	94 (36%)	9 (39%)	2 (67%)	27 (36%)	44 (35%)		
Severely abnormal	121 (25%)	69 (27%)	7 (30%)	0 (0%)	18 (24%)	27 (21%)		
Unavailable	17	17	0	0	0			
EEG/aEEG background by expert reviewer							0.30	-
Predominantly normal	36 (24%)	-	2 (9%)	-	-	34 (27%)		
Normal/Discontinuous	73 (49%)	-	15 (65%)	-	-	58 (46%)		
Predominantly discontinuous	11 (7%)	-	2 (9%)	-	-	8 (6%)		
Discontinuous/Severely abnormal	17 (11%)	-	3 (13%)	-	-	14 (11%)		
Predominantly severely abnormal	13 (9%)	-	1 (4%)	-	-	13 (10%)		
Unknown	350	271	-	3	76	-		
**Median BSN**								
Onset	-	-	-	57 (37, 76)	65 (37, 84)	67 (43, 82)	-	0.58
12-24 hours	-	-	-	38 (30, 51)	66 (38, 85)	68 (48, 83)	-	0.98
36-48 hours	-	-	-	46 (44, 51)	75 (47, 89)	73 (50, 83)	-	0.42
60-72 hours	-	-	-	45 (45, 68)	69 (39, 86)	74 (56, 83)	-	0.52
**Outcomes**								
Severe NDI or death	119 (24%)	65 (24%)	5 (22%)	-	21 (28%)	28 (22%)	0.09	0.16
5-level ordinal outcome							0.14	0.52
No NDI	236 (49%)	116 (46%)	11 (48%)	-	36 (47%)	73 (57%)		
Mild NDI	56 (12%)	27 (11%)	4 (17%)	-	9 (12%)	16 (13%)		
Moderate NDI	69 (14%)	46 (18%)	3 (13%)	-	10 (13%)	10 (7.9%)		
Severe NDI	54 (11%)	30 (12%)	3 (13%)	-	10 (13%)	11 (8.7%)		
Death	65 (14%)	35 (14%)	2 (8.7%)	-	11 (14%)	17 (13%)		
Unknown	20	17	0	3	0	0		

Data is displayed as median (IQR) for continuous variables and n (percentage) for categorical variables
Group details are explained in Figure S1.

© 2025 Cornet MC et al. *JAMA Network Open*.

P-values were calculated using chi-squared for categorical variables, and Wilcoxon rank-sum test for continuous variables.

© 2025 Cornet MC et al. *JAMA Network Open*.

eTable 2. Characteristics of Infants Classified as Having Severe NDI

Patient	BSID Cognitive	BSID Motor	BSID Language	CP	Modified GMFCS
1	Severe	Normal	Severe	No CP	0
2	Severe	Moderate	Moderate	No CP	0
3	Severe	Moderate	Moderate	No CP	0
4	Severe	Severe	Severe	No CP	0
5	Severe	Severe	Severe	No CP	0
6	Severe	Severe	Severe	No CP	0
7	Severe	Severe	Severe	No CP	1
8	Severe	Severe	Severe	Diparesis	1
9	Severe	Severe	Severe	Diparesis	1
10	Severe	Severe	Severe	Hemiparesis	1
11	Severe	Severe	NA	Quadriparesis	2
12	Severe	Severe	Severe	Quadriparesis	1
13	Severe	Severe	Severe	Quadriparesis	4
14	Severe	Severe	Severe	Quadriparesis	5
15	Severe	Severe	Severe	Quadriparesis	5
16	Severe	Severe	Severe	Quadriparesis	5
17	Severe	Severe	Severe	Quadriparesis	5
18	Severe	Severe	Severe	Quadriparesis	2
19	Severe	Severe	Moderate	Quadriparesis	1
20	Moderate	Severe	Normal	Quadriparesis	2
21	Mild	Severe	Mild	Quadriparesis	3

Classification of BSID scores: Normal≥90; Mild 85-89; Moderate 70-84; Severe<70

© 2025 Cornet MC et al. *JAMA Network Open*.

eTable 3. AUROC for 5 Imputed Datasets, including all 226 Infants with EEG Processed Through BABA, including those with missing BSN at all timepoints.

Variables included in model	203 Obs (no imputation)	226 observations				
		Imp 1	Imp 2	Imp3	Imp 4	Imp 5
1.BSN Onset	0.85 [0.79, 0.92]	0.86 [0.79, 0.92]	0.85 [0.79, 0.92]	0.84 [0.78, 0.91]	0.85 [0.79, 0.91]	0.86 [0.80, 0.92]
2.BSN 24h	0.86 [0.78, 0.93]	0.84 [0.77, 0.91]	0.84 [0.76, 0.91]	0.83 [0.75, 0.91]	0.85 [0.78, 0.92]	0.83 [0.75, 0.90]
3. BSN 48h	0.85 [0.78, 0.93]	0.84 [0.77, 0.91]	0.84 [0.77, 0.91]	0.83 [0.76, 0.91]	0.83 [0.76, 0.91]	0.80 [0.71, 0.88]
4. BSN 72h	0.87 [0.81, 0.94]	0.85 [0.79, 0.92]	0.86 [0.80, 0.92]	0.83 [0.76, 0.90]	0.86 [0.80, 0.92]	0.84 [0.77, 0.90]
5. BSN Onset+24+48+72h	0.92 [0.87, 0.97]	0.90 [0.85, 0.96]	0.90 [0.84, 0.95]	0.89 [0.83, 0.95]	0.90 [0.85, 0.96]	0.89 [0.84, 0.95]
6. Expert review	0.90 [0.81, 0.98]	0.89 [0.81, 0.97]	0.89 [0.81, 0.97]	0.89 [0.81, 0.97]	0.89 [0.81, 0.97]	0.89 [0.81, 0.97]
7. Clinical variables	0.79 [0.70, 0.87]	0.79 [0.71, 0.86]	0.79 [0.71, 0.86]	0.79 [0.71, 0.86]	0.79 [0.71, 0.86]	0.79 [0.71, 0.86]
8. Clinical + BSN Onset	0.88 [0.82, 0.94]	0.88 [0.83, 0.94]	0.88 [0.82, 0.94]	0.88 [0.81, 0.94]	0.88 [0.82, 0.94]	0.88 [0.82, 0.94]
9. Clin + BSN median overall	0.90 [0.84, 0.97]	0.90 [0.84, 0.97]	0.90 [0.84, 0.97]	0.90 [0.84, 0.97]	0.90 [0.84, 0.97]	0.90 [0.84, 0.97]
10. Clinical + BSN Onset+24+48+72h	0.93 [0.88, 0.98]	0.90 [0.85, 0.96]	0.90 [0.84, 0.96]	0.89 [0.83, 0.95]	0.91 [0.85, 0.96]	0.90 [0.85, 0.95]

© 2025 Cornet MC et al. *JAMA Network Open*.

eFigure 1. Maximal, Median, and Minimal BSN Values Based on EEG Continuity Scoring by Expert Reviewers at Each Time Point

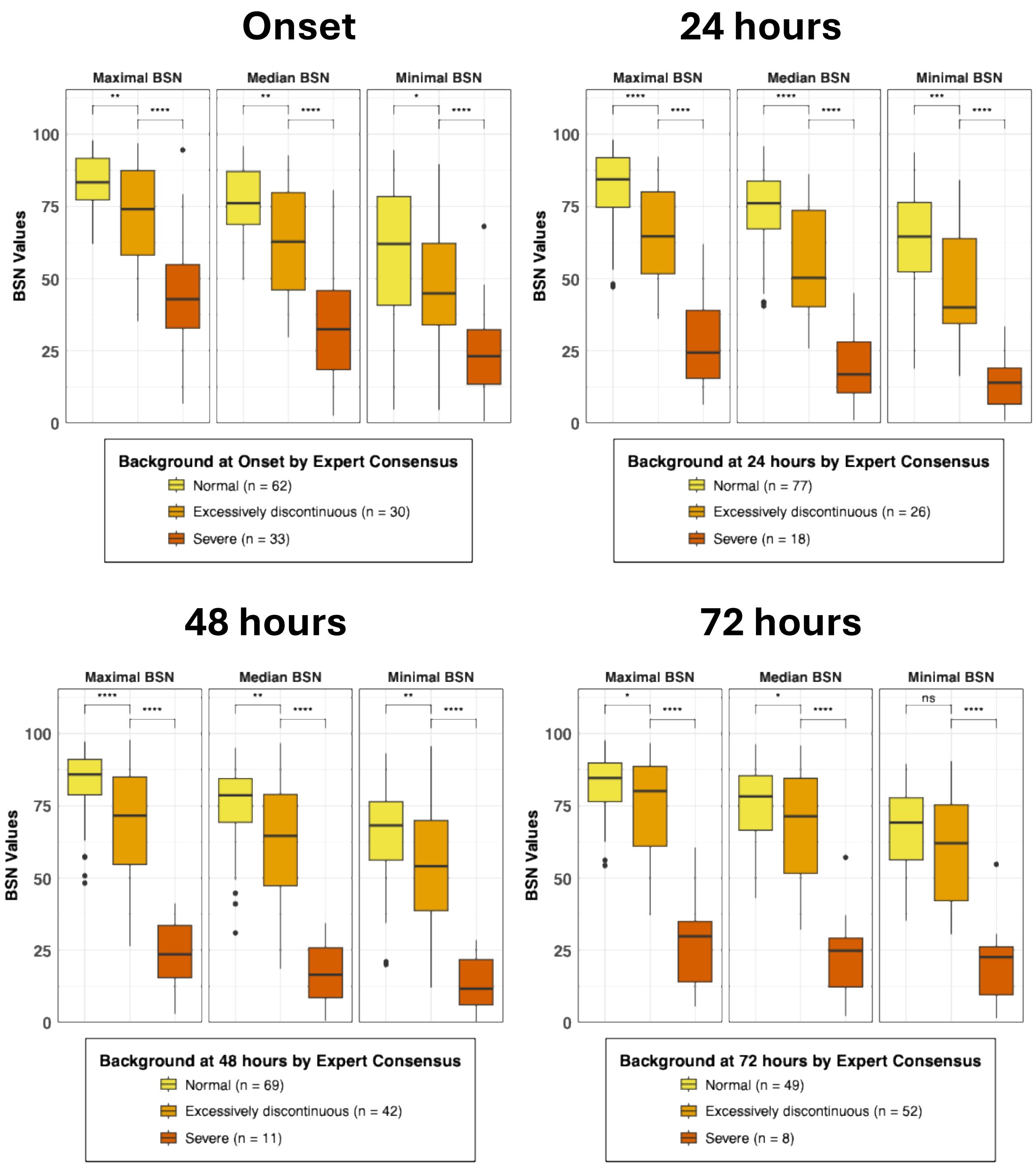

© 2025 Cornet MC et al. *JAMA Network Open*.

eFigure 2. ROC Curves Projecting Severe NDI or Death for Minimal and Maximal BSN Values

A

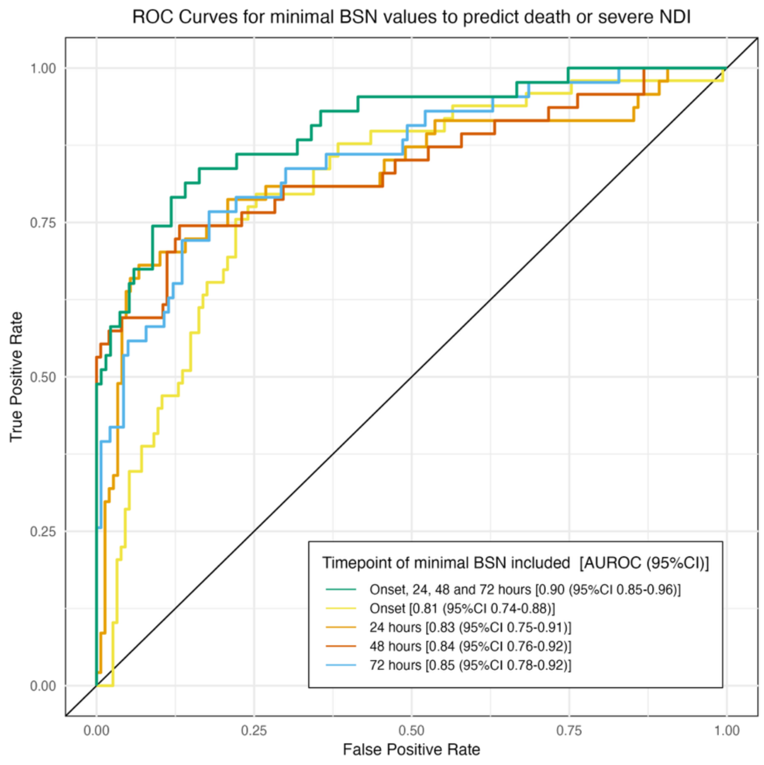

B

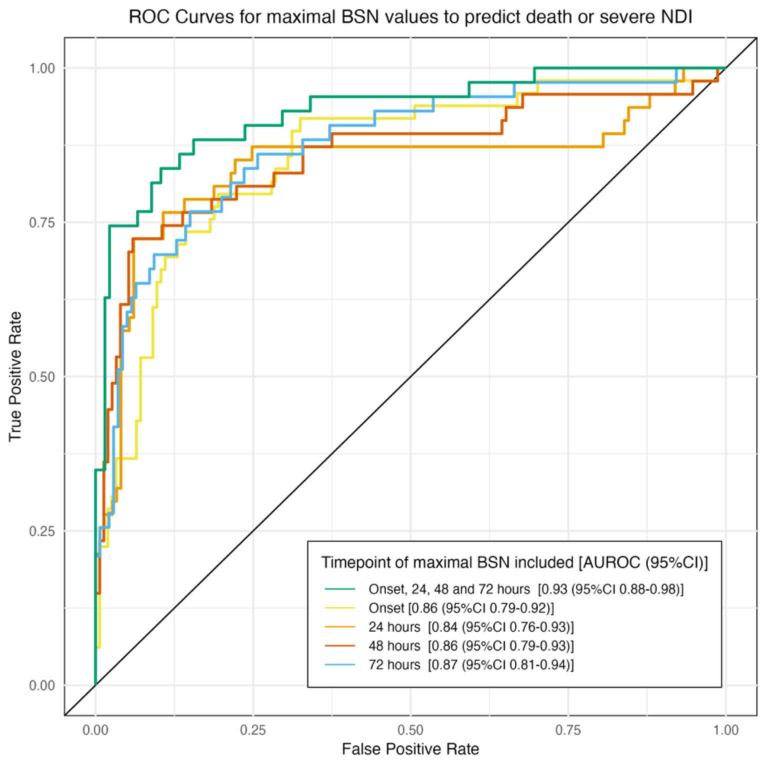

© 2025 Cornet MC et al. *JAMA Network Open*.

eFigure 3. Neurodevelopmental Outcomes Based on Minimal and Maximal BSN Score at Each Epoch

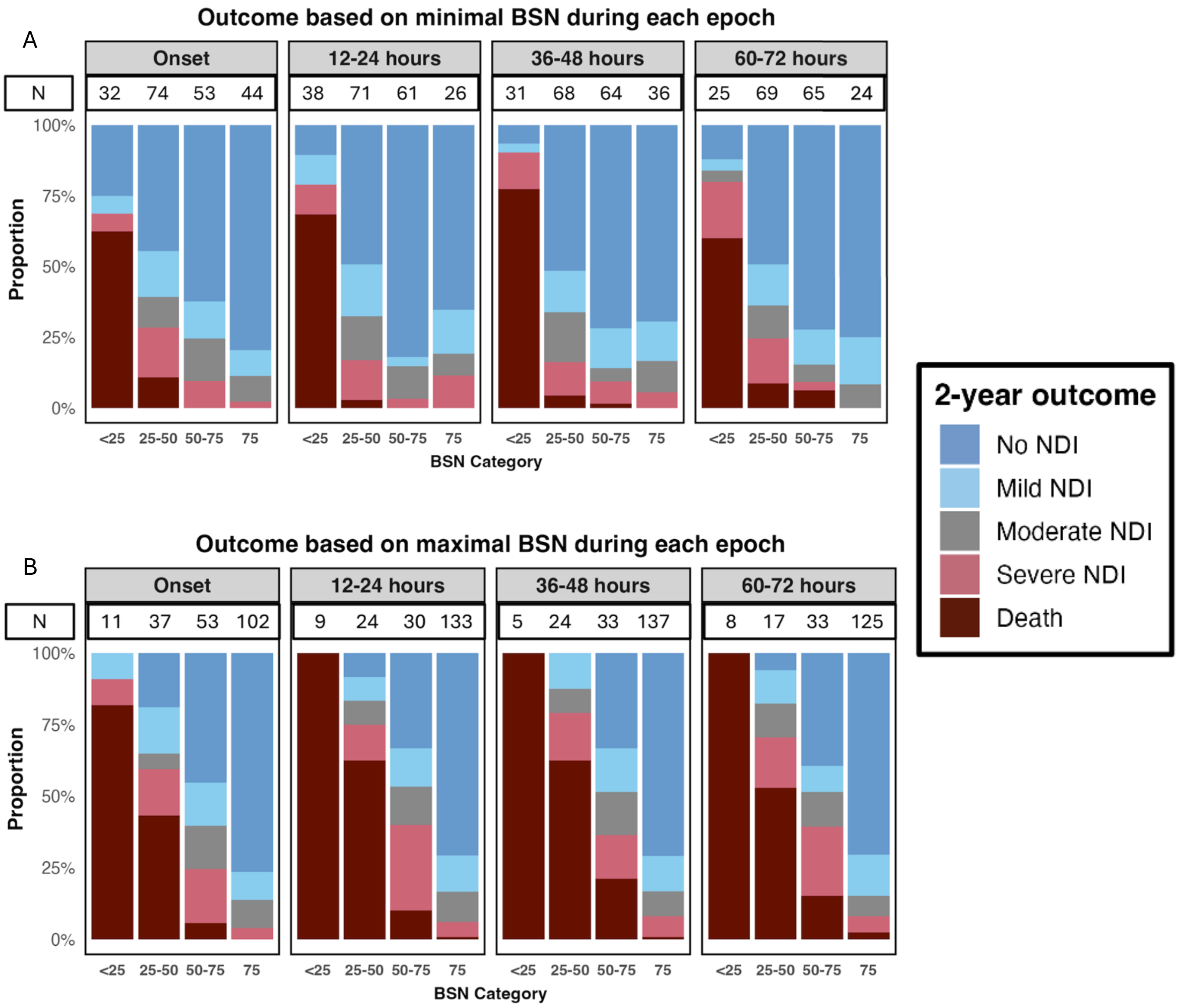

© 2025 Cornet MC et al. *JAMA Network Open*.
