# Supplementary material for: Automated EEG Background Analysis and 2-Year Outcomes in Neonatal Hypoxic-Ischemic Encephalopathy
Source: JAMA Netw Open. 2025 Dec 16;8(12):e2548321. doi: 10.1001/jamanetworkopen.2025.48321 (PMC12709380; doi:10.1001/jamanetworkopen.2025.48321)
Supplement: Supplement 2. — Data Sharing Statement [file jamanetwopen-e2548321-s002.pdf]

Data Sharing Statement

Data
Data available: No

Additional Information
Explanation for why data not available: Part of the data is available upon reasonable request to the study authors or on the NINDS Data repository.
